# *In vitro* and *in vivo* study of epigallocatechin-3-gallate-induced apoptosis in aerobic glycolytic hepatocellular carcinoma cells involving inhibition of phosphofructokinase activity

**DOI:** 10.1038/srep28479

**Published:** 2016-06-28

**Authors:** Sainan Li, Liwei Wu, Jiao Feng, Jingjing Li, Tong Liu, Rong Zhang, Shizan Xu, Keran Cheng, Yuqing Zhou, Shunfeng Zhou, Rui Kong, Kan Chen, Fan Wang, Yujing Xia, Jie Lu, Yingqun Zhou, Weiqi Dai, Chuanyong Guo

**Affiliations:** 1 Department of Gastroenterology, Shanghai Tenth People’s Hospital, Tongji University School of Medicine, Shanghai 200072, China; 2 The Shanghai Tenth Hospital School of Clinical Medicine of Nanjing Medical University, Shanghai 200072, China; 3 The School of Medicine of Soochow University, Suzhou 215006, China

## Abstract

Glycolysis, as an altered cancer cell-intrinsic metabolism, is an essential hallmark of cancer. Phosphofructokinase (PFK) is a metabolic sensor in the glycolytic pathway, and restricting the substrate availability for this enzyme has been researched extensively as a target for chemotherapy. In the present study, we investigated that the effects of epigallocatechin-3-gallate (EGCG), an active component of green tea, on inhibiting cell growth and inducing apoptosis by promoting a metabolic shift away from glycolysis in aerobic glycolytic hepatocellular carcinoma (HCC) cells. EGCG modulated the oligomeric structure of PFK, potentially leading to metabolic stress associated apoptosis and suggesting that EGCG acts by directly suppressing PFK activity. A PFK activity inhibitor enhanced the effect, while the allosteric activator reversed EGCG-induced HCC cell death. PFK siRNA knockdown-induced apoptosis was not reversed by the activator. EGCG enhanced the effect of sorafenib on cell growth inhibition in both aerobic glycolytic HCC cells and in a xenograft mouse model. The present study suggests a potential role for EGCG as an adjuvant in cancer therapy, which merits further investigation at the clinical level.

Unlike normal differentiated cells, cancer cells are highly dependent on aerobic glycolysis even under normoxia, in a phenomenon called the “Warburg effect”^1^,^2^,^3^. Aerobic glycolysis is an inefficient way to generate adenosine 5′-triphosphate (ATP), by converting pyruvate to lactate rather than totally oxidizing it through the Krebs cycle^4^. This constitutes an advantage for tumor growth for two main reasons: first, cancer cells can survive conditions of fluctuating oxygen tension that would be lethal for cells that rely on oxidative phosphorylation (OXPHOS) to generate ATP because of the variable hemodynamics of distant blood vessels^4^; and second, lactate as the principal end product of aerobic glycolysis, generates an acid environment that favors tumor invasion and suppresses anticancer immune effectors^5^,^6^,^7^.

Aerobic glycolysis itself is controlled by the activity of three key allosteric enzymes: hexokinase (HK), phosphofructokinase (PFK) and pyruvate kinase (PK)^8^. Of the three rate limiting enzymes of the pathway, isoforms of PFK are considered the pacemakers of glycolysis^9^. PFK1 catalyzes the MgATP-dependent phosphorylation of fructose-6-phosphate (F6P) to form ADP and fructose-1,6-bisphosphate (F1,6BP)^9^, and PFK2 produces fructose-2,6-bisphosphate (F2,6BP), which is the most potent allosteric activator of PFK^10^. In human carcinomas, including hepatocellular carcinoma (HCC), PFK is highly expressed and activated to produce the additional energy required to support accelerated growth^11^,^12^. A recent study demonstrated that apoptosis is closely related to glycolysis based on the association of the pro-apoptotic protein Bad with PFK^13^. PFK is a potentially important target to deprive cancer cells from essential energy and substrates for macromolecular synthesis and proliferation while allowing normal cells to survive^8^.

Green tea is an extremely popular beverage worldwide that has long been associated with health benefits, including chemo-preventive effects^14^. Epigallocatechin-3-gallate (EGCG) is the most effective compound in green tea; it has strong chemo-preventive effects and has been suggested as a potential chemotherapeutic agent against cancers of the skin (UV radiation and chemically induced)^15^, lung^16^, breast^17^, colon^18^, liver^19^, prostate^20^, and other sites^21^,^22^. Studies on a variety of cancer cell lines, including HeLa, A549, and MCF-7, have shown that the chemo-preventive effect of EGCG is mediated by the induction of apoptosis and cell cycle arrest, and the inhibition of angiogenesis, metastasis and migration^23^. Different mechanisms have been proposed to explain the cancer-preventive effect of EGCG^24^,^25^ in addition to its widely known antioxidant potential^26^, the upregulation of tumor suppressor genes such as p53^27^, and the modulation of cell signaling pathways, such as the inhibition of nuclear factor-κB (NF-κB)^28^, mitogen-activated protein kinase (MAPK)^29^, epidermal growth factor receptor (EGFR)^18^, and insulin-like growth factor (IGF)^30^. Recent evidence suggests the involvement of the JAK/STAT3 signaling pathway in the multiple therapeutic effects of EGCG^31^,^32^.

The effect of EGCG on the expression and activity of PFK during the metabolic transformation of HCC cells has not been investigated in detail. In the present study, we show that the metabolic phenotype of HCC cells is characterized by glucose to lactate conversion and suppressed oxidative activity. EGCG inhibits glycolysis and induces apoptosis in HCC cells. Further investigation of the underlying mechanism showed that EGCG inhibited the expression and activity of PFK. In addition, EGCG improved the resistance of aerobic glycolytic HCC cells to the multikinase inhibitor sorafenib, the standard first-line systemic drug that can slightly prolong the survival of HCC patients. The results of the present study improve our understanding of the mechanisms underlying the effect of EGCG on tumor proliferation and metabolism, and may help identify effective treatments for patients with HCC.

## Results

### Glycolysis and glucose uptake in HCC cell lines

**Figure 1:**
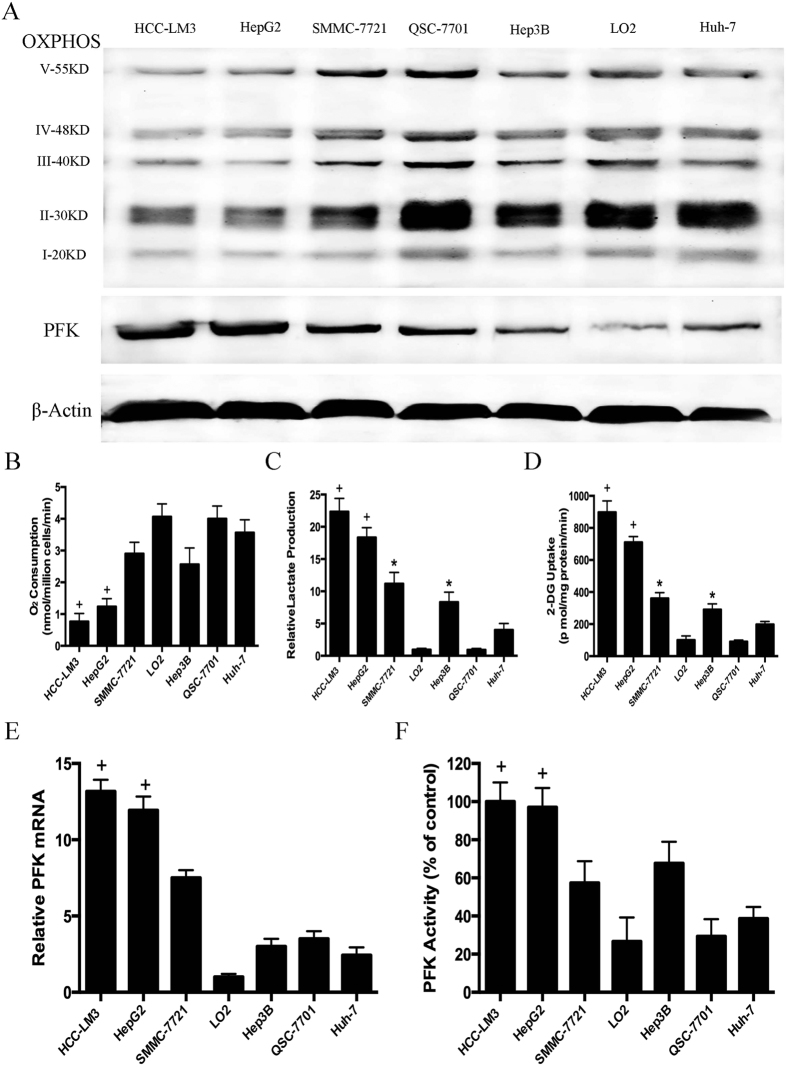
Metabolic features and expression and activity of phosphofructokinase (PFK) in hepatocellular carcinoma (HCC) cell lines. (**A**) Western blot analysis of oxidative phosphorylation (OXPHOS) and PFK in total cell extracts from HCC cells and normal liver cells. β-actin was used as a loading control. (**B**) O_2_ consumption in the indicated cell lines (nmol O_2_/million cells/min) was tested by a Clark-type oxygen electrode, which detected the concentration of dissolved oxygen in a closed chamber over time. (**C**,**D**) Normalized lactate production and 2-DG uptake in HCC cells (SMMC-7721, Hep3B, HepG2, HCC-LM3, Huh-7) and normal hepatic cells (QSG-7701 and LO2) within 24 h of culture under normoxic conditions. The lactate production was normalized to the level of LO2 cell line. (**E**) qRT-PCR analysis of PFK expression in HCC and normal liver cells. The mRNA expression was normalized to the level of LO2 cell line. (**F**) PFK activity was measured by the spectrophotometric method. PFK activity was normalized to the level of the HCC-LM3 cell line. Plotted values represent the mean ± standard error of three independent experiments (n = 3) (*P < 0.05; ^+^P < 0.01).

Most cancer cells, especially those with the most aggressive phenotypes, show a substantial uncoupling of glycolysis from OXPHOS with the consequent production of high levels of lactate^3^,^10^. The byproducts of OXPHOS and O_2_ consumption along with lactate production were compared between HCC cell lines and two normal hepatic cell lines (QSG-7701 and LO2)^33^ characterized by differences in cellular metabolism (Fig. 1A–C). The concentration of lactate in the cell culture supernatant was significantly higher in HCC-LM3, SMMC-7721, Hep3B and HepG2 cells than in Huh-7, QSG-7701 and LO2 cells. OXPHOS metabolism-correlated proteins, denoted as complexes I/II/III/IV/V in the electron transport chain, were markedly decreased in the representative aerobic glycolytic HCC cell lines (HCC-LM3, SMMC-7721, Hep3B and HepG2), compared with their levels in the low-glycolytic HCC cell line (Huh-7) and healthy cells (QSG-7701 and LO2). O_2_ consumption, which reflects the level of OXPHOS metabolism, was lower in representative aerobic glycolytic HCC cells (HCC-LM3 and HepG2) than in the other cells examined (Fig. 1C), indicating that the rate of aerobic glycolysis was higher in HCC cell lines than in normal cells.

The low efficiency of energy production of anaerobic glycolysis requires a higher consumption of glucose. Determination of glucose uptake in the different cell lines (Fig. 1D) showed a higher rate of glucose uptake in HCC-LM3 and HepG2 cells (898 vs. 710 pmol/mg/min of 2-deoxy-D-glucose (2-DG), respectively) than in healthy cells, followed by SMMC-7721 and Hep3B cells, whereas Huh-7 cells showed no increase in lactate production or glucose uptake. Consistent with the lactate production, these results further demonstrated that HCC cell lines showed an increased rate of aerobic glycolysis compared to healthy cells. Taken together, HCC-LM3 and HepG2 were cell lines with the highest glycolytic rate and were selected for further investigation. The low-glycolytic HCC cell line Huh-7 and the normal hepatic cell line LO2 were used as controls.

### Expression and activity of PFK in HCC cell lines

Because PFK is an important enzyme in aerobic glycolysis^8^, PFK expression was determined by western blotting and RT-PCR in two normal hepatic cell lines (QSG-7701 and LO2) and five HCC cell lines (HCC-LM3, SMMC-7721, Hep3B, HepG2, and Huh-7). As shown in Fig. 1A,E, PFK mRNA and protein levels were high in HCC-LM3 and HepG2 cells, followed by SMMC-7721 and Hep3B cells. PFK activity was assessed using a spectrophotometric method. As shown in Fig. 1F, PFK was highly activated in HCC-LM3 and HepG2 cells. The increased expression and activity of PFK in HCC cell lines with a high rate of aerobic glycolysis indicated that PFK is an essential metabolic sensor in the glycolytic pathway.

However, although the mRNA and protein expression of PFK was relatively lower in LO2 cells than in QSG-7701 cells (Fig. 1A,E), PFK activity was comparable in the two healthy cells (Fig. 1F), demonstrating that the activity of PFK plays a more significant role in the glycolytic process than its expression.

### The compound EGCG inhibits glycolysis, especially the activity of PFK in aerobic glycolytic HCC cell lines

**Figure 2:**
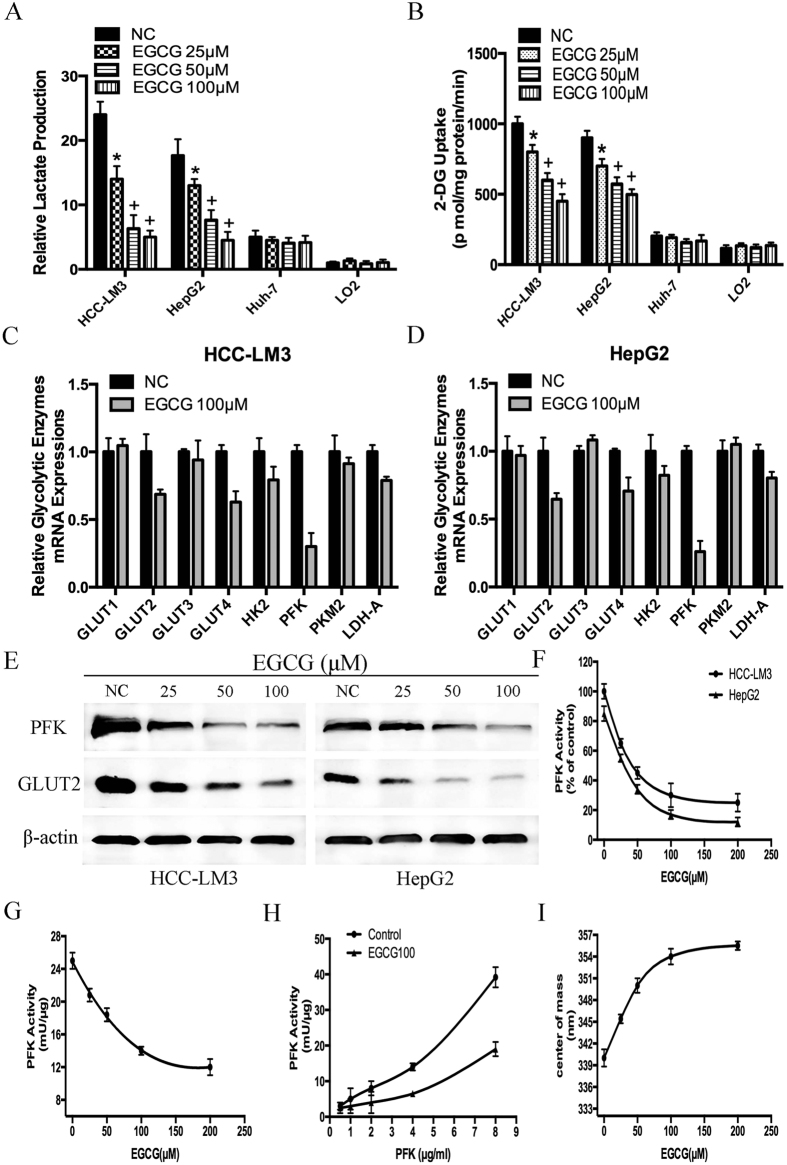
Effects of epigallocatechin-3-gallate (EGCG) on HCC cells glucose consumption, lactate production and PFK expression and activity. (**A**,**B**) Relative lactate production and 2-DG uptake from HCC cell lines (HCC-LM3, HepG2 and Huh-7) and normal hepatic cells (LO2) in the absence or presence of EGCG (25, 50, and 100 μM) within 24 h of culture under normoxic conditions. (**C**,**D**) qRT-PCR analysis of the effect of EGCG (100 μM) on the expression of genes associated with glycolysis in HCC cells. (**E**) Western blot analysis of EGCG (0, 25, 50, and 100 μM) on the protein expression of PFK and GLUT2 in HCC-LM3 and HepG2 cells. β-actin was used as a loading control. (**F**) Spectrophotometric analysis of the effect of EGCG (0, 25, 50, 100 and 200 μM) on PFK activity in HCC-LM3 and HepG2 cells. (**G**) Purified PFK activity was evaluated as described through the radiometric assay, in the presence of the desired concentrations of EGCG (25, 50, 100 and 200 μM). (**H**) PFK titration curve of enzyme-specific activity in the absence and presence of 100 μM EGCG. (**I**) Purified PFK was incubated for 1 h in the presence of the various concentrations of EGCG (0, 25, 50, 100 and 200 μM). The treated enzyme was used to evaluate its quaternary structure, assessing the center of mass of the intrinsic fluorescence spectra. Plotted values represent the mean ± standard error of three independent experiments (n = 3) (*P < 0.05; ^+^P < 0.01).

Since EGCG suppresses aerobic glycolysis in pancreatic^34^ and tongue^35^ cancer cells, we examined the ability of EGCG to induce similar changes in HCC cell lines. Our data showed that EGCG decreased lactate concentration in HCC-LM3 and HepG2 cells in a dose dependent manner compared with that in the untreated control group (Fig. 2A). Glucose uptake showed a similar pattern as that of lactate production in HCC-LM3 and HepG2 cells treated with EGCG (Fig. 2B). By contrast, EGCG treatment had no effect on lactate levels or glucose uptake in the low-glycolytic Huh-7 cells and healthy LO2 cells (Fig. 2A,B). These data suggested that EGCG treatment at doses of up to 100 μM only inhibited glycolysis in cancer cells with a high rate of aerobic glycolysis (HCC-LM3 and HepG2 cells) but not in low-glycolytic cells (Huh-7 and LO2 cells).

To examine the mechanism underlying the effect of EGCG on inhibiting glycolysis in aerobic glycolytic HCC cells, the effects of EGCG on the expression of several key glycolytic enzymes, including GLUT1, GLUT2, GLUT3, GLUT4, HK2, PFK, PKM2, and LDH-A were assessed in HCC-LM3 and HepG2 cells using qRT-PCR. Among these, PFK was the most down-regulated enzyme at the mRNA level in both HCC-LM3 and HepG2 cells treated with 100 μM EGCG (Fig. 2C,D). Western blot analysis indicated that HCC-LM3 and HepG2 cells treated with EGCG for 24 h showed a significant decrease in PFK protein expression in a dose-dependent manner (Fig. 2E).

Previous studies showed that the activity of PFK is not correlated with its expression level^36^. Therefore, the spectrophotometric method was used to measure PFK activity. Similar to the effects of EGCG on lactate reduction and glucose consumption, this compound decreased PFK activity (Fig. 2F) by approximately 55% and 63% in HCC-LM3 and HepG2 cells, respectively, when used at a concentration of 50 μM. These data indicated that the effect of EGCG on glycolysis inhibition was mainly mediated by the inhibition of the activity of PFK. Next, to investigate whether EGCG could be acting directly on PFK, the activity of the purified enzyme was analyzed in the presence of different concentrations of EGCG. The radiometric assay showed that in the presence of the desired concentrations of EGCG, the activity of PFK was inhibited in a concentration-dependent manner (Fig. 2G), with an IC_50_ of 124.16 μM.

There are two major oligomeric conformations of PFK: the quite inactive dimers and the fully active tetramers^37^. The oligomers are shifted to more complex structures at higher concentrations of the enzyme, and the specific activity of the enzyme increases when the tetramers are stabilized at high PFK concentrations^38^. We calculated the transition between conformations of low specific activity (dimers) and high specific activity (tetramers) in a PFK concentration curve of the enzyme-specific activity. As shown in Fig. 2H, in the presence of EGCG, the specific activity was not altered up to 4 μg/ml PFK, which was different from the control experiments, in which the specific activity increased gradually with increasing PFK concentrations. This suggested that high enzyme concentrations promoted the formation and stabilization of more complex oligomeric forms of the enzyme, thus preventing the inhibitory effects of EGCG.

The effects of EGCG on the oligomeric structure of PFK were assessed by measuring the center of mass of the intrinsic fluorescence spectra of PFK. The inactive dimers (lower energy, higher wavelength) were identified in the red region of the spectrum, whereas the fully active tetramers (higher energy, lower wavelength) were shown in the blue region of the spectrum^39^,^40^. The results showed that EGCG promoted the shift from blue to red of the center of mass (Fig. 2I), which occurred in parallel to the inhibition of enzyme activity (Fig. 2H). This strongly suggested that EGCG stabilized the dimeric conformation of PFK, inhibiting the enzyme. Taken together, these results indicated that the effect of EGCG on glycolysis inhibition was mainly mediated by decreasing the activity of PFK through its stabilization in the inactive oligomeric conformation.

Analysis of the potential effect of EGCG on other glycolytic proteins showed that at a dose of 50 μM, EGCG down-regulated the protein expression of GLUT2 in HCC-LM3 and HepG2 cells (Fig. 2E), suggesting that EGCG may inhibit glycolysis at a broad level in aerobic glycolytic HCC cells.

### The compound EGCG inhibits proliferation, arrests cells in S Phage and induces apoptosis

**Figure 3:**
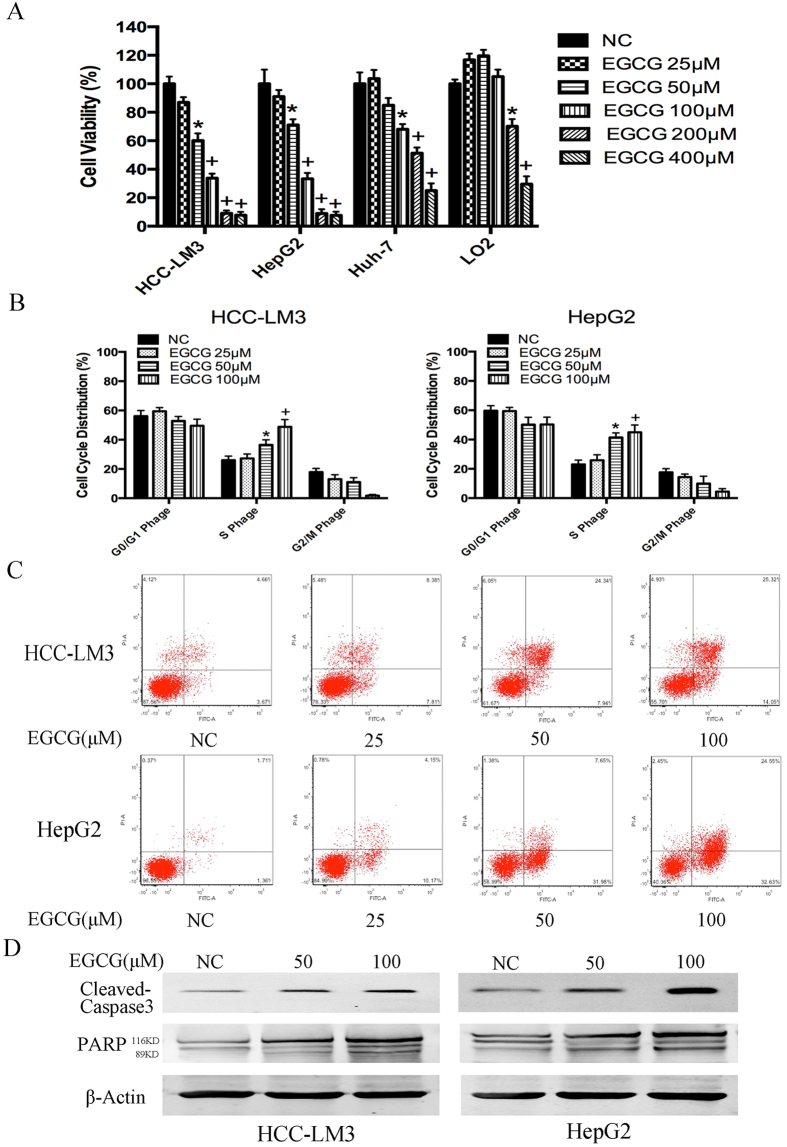
Effects of EGCG on HCC cell proliferation inhibition, S phage arrest, and apoptosis induction. (**A**) HCC cells were cultured with or without EGCG (25, 50, 100, 200 and 400 μM) for 24 h and cell viability was examined using the CCK-8 method. (**B**) EGCG induced cell cycle arrest in HCC-LM3 and HepG2 cells. Cells were treated with the indicated concentrations of EGCG for 24 h, stained with propidium iodide, and analyzed by flow cytometry. (**C**) HCC-LM3 and HepG2 cells were treated with different concentrations of EGCG, and the cell apoptosis rate was examined by flow cytometry. (**D**) Western blot analysis of cleaved-caspase 3 and PARP in HCC cells treated with EGCG (0, 50, and 100 μM). β-actin was used as a loading control. Plotted values represent the mean ± standard error of three independent experiments (n = 3) (*P < 0.05; ^+^P < 0.01).

EGCG-suppressed glycolysis in tumor cells leads to the inhibition of proliferation in multiple cancers^41^. Therefore, we examined the effect of EGCG on the proliferation of HCC cell lines. HCC-LM3, HepG2, Huh-7 and LO2 cells were treated with various concentrations of EGCG for proliferation test. Results showed that cell proliferation was inhibited in a time-dependent manner in HCC-LM3 and HepG2 cells (Fig. S1). With EGCG treatment for 24 h, cell proliferation rate was already significantly inhibited in a dose-dependent manner in three HCC cell lines, albeit to varying degrees (Fig. 3A). Thus, 24 h was chosen as the proper time point for further study. The IC50 of EGCG for the inhibition of cell proliferation in HCC-LM3, HepG2, Huh-7 and LO2 cells was 65.81, 74.04, 191.03 and 288.31 μM, respectively. Therefore, dosage at 25, 50 and 100 μM were selected for further research.

There are several reasons that may lead to proliferation inhibition, and arresting cells at a certain phage of cell cycle is a significant one^20^,^42^. Therefore, the effect of EGCG on cell cycle distribution was detected. After EGCG treatment for 24 h, the proportion of S Phage was significantly increased in HCC-LM3 and HepG2 cells (Fig. 3B). EGCG at a concentration of 50 and 100 μM significantly arrested cells in S Phage in HCC-LM3 cells (36.47%, 48.82% *vs*. 26.02%, P = 0.038, P = 0.0079, respectively) and HepG2 cells (41.48%, 44.94% *vs*. 23.06%, P = 0.017, P = 0.0021, respectively) compared to untreated cells.

In addition to cell proliferation inhibition and S Phage arresting, a major effect of EGCG treatment is the induction of apoptosis in various types of cancer cells^15^,^31^. Therefore, we investigated whether EGCG treatment induced apoptosis in HCC cells. Annexin-V/PI double staining indicated that EGCG at a concentration of 50 and 100 μM significantly induced apoptosis in HCC-LM3 cells (32.28%, 39.37% *vs*. 8.33%, P = 0.048, P = 0.0091, respectively) and HepG2 cells (39.60%, 57.18% *vs*. 3.07%, P = 0.013, P = 0.0016, respectively), albeit to varying degrees (Fig. 3C). EGCG at a concentration of 50 μM significantly induced apoptosis in HepG2 cells compared to untreated cells, whereas concentrations above 100 μM were required to achieve a similar effect in HCC-LM3 cells. Consistent with the apoptosis staining results, western blotting revealed a significant up-regulation of active caspase-3 and cleaved PARP (poly ADP-ribose polymerase) in HCC-LM3 and HepG2 cells treated with 50 μM of EGCG (Fig. 3D). These results indicated that EGCG enhanced the apoptosis-induced functions in aerobic glycolytic HCC cells.

### Citrate-mediated PFK inactivation in aerobic glycolytic HCC cells enhances EGCG-induced apoptosis

**Figure 4:**
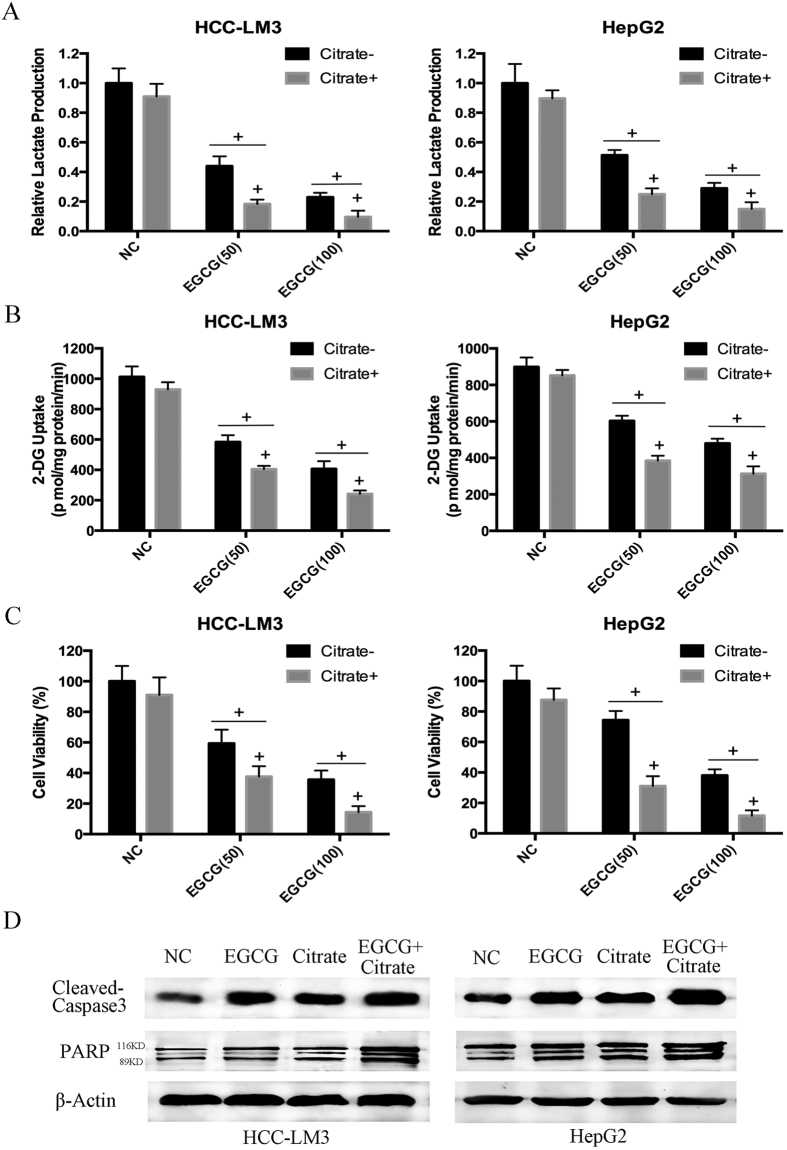
Citrate enhanced the effect of EGCG on HCC cells. (**A**–**C**) HCC-LM3 and HepG2 cells were treated with EGCG (50 and 100 μM) with or without citrate (5 mM) for 24 h. At the time points indicated, the following measurements were performed: lactate production (**A**), 2-DG uptake (**B**), cell proliferation rate (**C**). (**D**) Western blot analysis of cleaved-caspase 3 and PARP of HCC cells treated with EGCG (50 μM), citrate (5 mM), or combination for 24 h. β-actin was used as a loading control. Plotted values represent the mean ± standard error of three independent experiments (n = 3) (^+^P < 0.01).

Since the degree of cell viability inhibition and apoptosis enhancement by EGCG (Fig. 3) was highly correlated with decreased PFK activity (Fig. 2F–I), lactate production (Fig. 2A), and glucose consumption (Fig. 2B), we hypothesized that EGCG-induced cell death was correlated to the inhibition of glycolysis, and PFK was a target of EGCG-induced cell death in HCC-LM3 and HepG2 cells. Because inhibitors of PFK activity could enhance the growth inhibitory and apoptosis enhancement effects of EGCG on aerobic glycolytic HCC cells, HCC-LM3 and HepG2 cells were treated with EGCG in the presence or absence of sodium citrate, a direct PFK activity inhibitor^43^. Citrate (5 mM) decreased both lactate production and glucose uptake in the presence or absence of EGCG in HCC-LM3 and HepG2 cells (Fig. 4A,B), demonstrating its glycolysis inhibiting effect. EGCG effectively suppressed the growth of both citrate- and citrate+ cells (Fig. 4C). The inhibitory effect of EGCG was stronger in citrate+ than in citrate- cells, suggesting that citrate promoted the cell apoptosis effect of EGCG. Western blot analysis showed that citrate enhanced the EGCG upregulation of active caspase-3 and cleaved-PARP in both HCC-LM3 and HepG2 cells (Fig. 4D). The enhancement effect of EGCG and the PFK activity inhibitor citrate indicated that EGCG induced apoptosis partly by suppressing HCC glycolysis via inhibiting PFK activity.

### EGCG enhanced apoptosis partly by suppressing HCC glycolysis via directly inhibiting PFK activity

**Figure 5:**
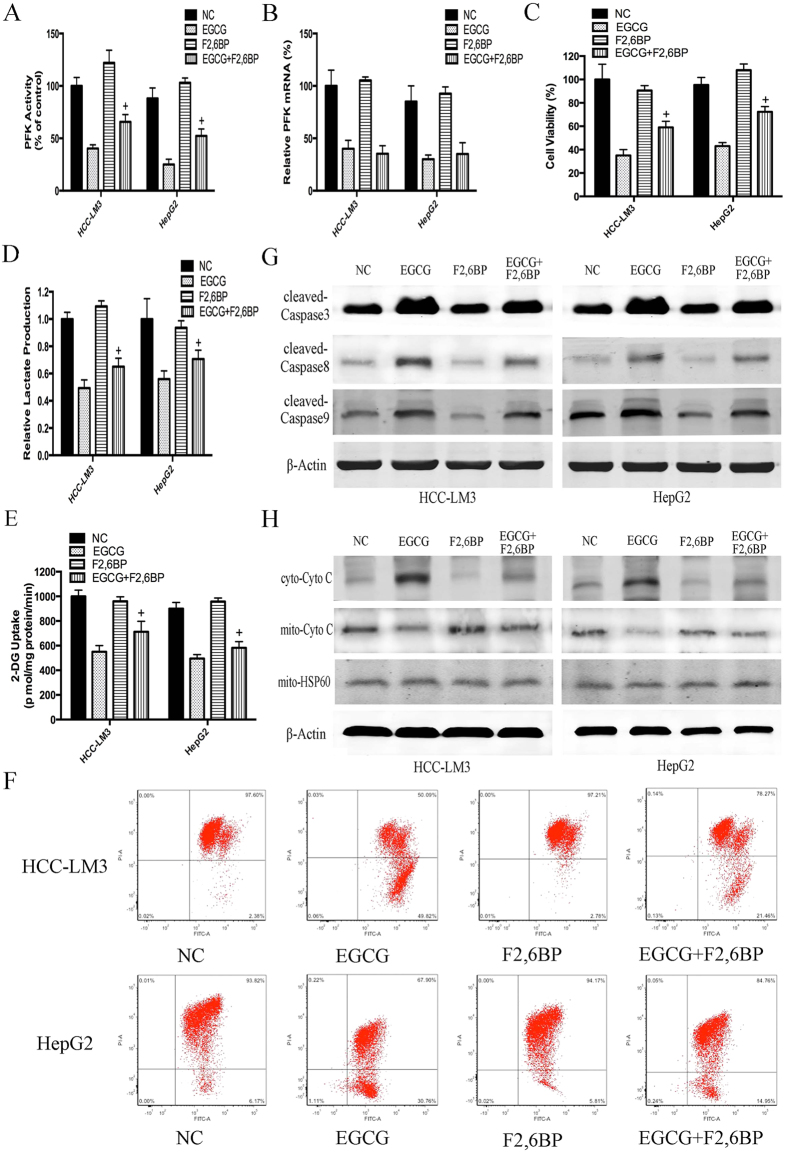
F2,6BP reversed the effect of EGCG on HCC cell glycolysis and apoptosis. (**A**–**E**) HCC-LM3 and HepG2 were treated with EGCG (100 μM) with or without F2,6BP (5  μM) for 24 h. At the time points indicated, the following measurements were performed: PFK activity (**A**), PFK mRNA expression (**B**), cell proliferation rate (**C**), lactate production (**D**), 2-DG uptake (**E**). (**F**) To measure changes in the Δψm, cells (5 × 10^4^) treated with EGCG with or without F2,6BP were stained with JC-1 (10 μg/ml) and analyzed by flow cytometry. (**G**,**H**) Western blot analysis of cleaved-caspase 3, cleaved-caspase 8, cleaved-caspase 9, mito-cyto c and cyto-cyto c in HCC-LM3 and HepG2 cells treated EGCG with or without F2,6BP for 24 h. Actin and hsp-60 served as loading controls. Plotted values represent the mean ± standard error of three independent experiments (n = 3) (^+^P < 0.01).

To further investigate whether EGCG-induced cell death was correlated to the suppression of glycolysis, F2,6BP, a potent allosteric activator of PFK was used at a concentration of 5 μM as previously reported^40^. We hypothesized that the activation of PFK by F2,6BP in aerobic glycolytic HCC cells would attenuate EGCG-induced apoptosis. As shown in Fig. 5A, F2,6BP significantly antagonized the inhibitory effect of EGCG on PFK activity. Moreover, F2,6BP effectively reversed the cell proliferation inhibitory effect of EGCG in aerobic glycolytic HCC cells (Fig. 5C) and also counteracted the glycolysis inhibitory effects of EGCG (Fig. 5D,E). However, interestingly, the expression of PFK did not differ in the presence or absence of F2,6BP (Fig. 5B), indicating that rather than promoting the synthesis of PFK, F2,6BP counteracted the cell proliferation inhibitory effect of EGCG by stimulating the activity of PFK, while EGCG inhibited both the expression and activity of PFK.

We next examined the mechanism by which EGCG induced apoptosis through the downregulation of PFK activity in HCC-LM3 and HepG2 cells. Decreased Δψm has been linked to apoptotic cell death^44^. To determine the effects of EGCG and F2,6BP on Δψm in apoptotic HCC-LM3 and HepG2 cells, flow cytometric analysis was performed. In PFK-activated cells treated with EGCG, Δψm was significantly increased by 21.46% and 14.95% in HCC-LM3 and HepG2 cells, respectively, compared with the rates in cells treated with EGCG alone (49.82% and 30.76%; P < 0.01, respectively; Fig. 5F), indicating that the effect of F2,6BP on inhibiting the EGCG-induced decrease in Δψm involves the modulation of PFK activity.

Induction of apoptosis requires the activation of caspases^45^. Therefore, western blot analysis was performed to investigate the involvement of caspase activation in EGCG-induced apoptosis. As shown in Fig. 5G, EGCG at a concentration of 100 μM combined with F2,6BP significantly attenuated EGCG-induced apoptosis in HCC-LM3 and HepG2 cells by inhibiting the activation of caspases 3, 8, and 9, confirming the involvement of PFK in EGCG-induced apoptosis.

**Figure 6:**
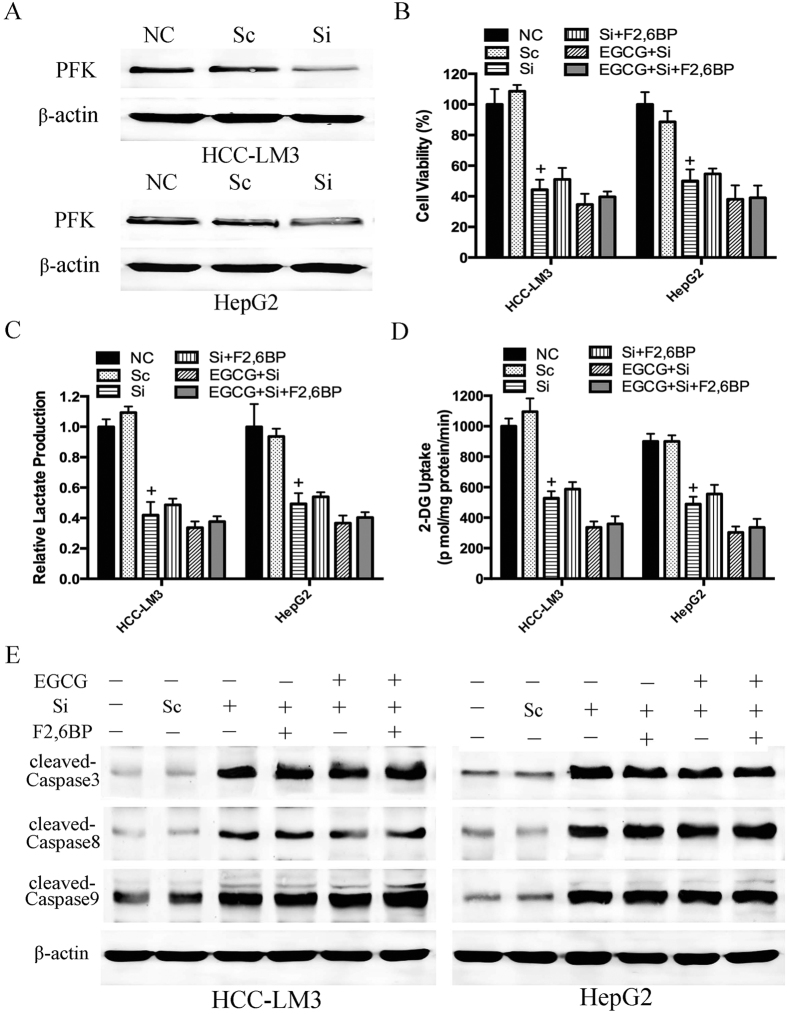
F2,6BP could not reverse the effect of EGCG on PFK siRNA knockdown HCC cell glycolysis and apoptosis. (**A**) Western blot analysis was used to detect the transduction efficiency of PFK in HCC cells. Actin served as a loading control. (**B**–**E**) PFK siRNA knockdown HCC-LM3 and HepG2 cells were treated with EGCG (100 μM) with or without F2,6BP (10 μM) for 24 h. At the time points indicated, the following measurements were performed: cell proliferation rate (**B**), lactate production (**C**), 2-DG uptake (**D**), western blot analysis of cleaved-caspase 3, cleaved-caspase 8, cleaved-caspase 9 and β-actin (**E**). Plotted values represent the mean ± standard error of three independent experiments (n = 3) (^+^P < 0.01).

Activation of caspase 9 occurs during the release of cytochrome c from mitochondria into the cytosol^46^. To test whether this release occurred in EGCG-induced apoptosis in HCC-LM3 and HepG2 cells, cytochrome c translocation was examined. As shown in Fig. 6B, cells treated with 100 μM EGCG showed translocation of cytochrome c from the mitochondria into the cytosol, and this effect was reversed by F2,6BP at a concentration of 5 μM. The decrease in cytosolic cyto c released from mitochondria to the cytosol in PFK-activated groups was consistent with the reduced depolarization effect of Δψm (Fig. 5F,H), demonstrating that activation of PFK by F2,6BP in aerobic glycolytic HCC cells attenuated EGCG-induced apoptosis, especially mitochondrial apoptosis.

To confirm the involvement of PFK in EGCG-induced liver cancer cell death, we performed siRNA-mediated knockdown of PFK in HCC-LM3 and HepG2 cells (Fig. 6A). As shown in Fig. 6B, cell viability decreased in PFK siRNA knockdown cells (the third bar), and this effect was not enhanced by treatment with EGCG (the fifth bar). By contrast, scramble siRNA (the second bar) showed no significant effect on proliferation as compared to that in the control. F2,6BP did not reverse the proliferation inhibitory effect in PFK siRNA knockdown cells with or without EGCG treatment (the fourth and sixth bars). The results of lactate production, glucose uptake and protein expression of caspases were consistent with the changes in cell viability (Fig. 6C–E), suggesting a direct involvement of PFK in EGCG-induced glycolysis inhibition and cell death.

### The pro-apoptotic protein Bad was involved in EGCG-induced apoptosis

**Figure 7:**
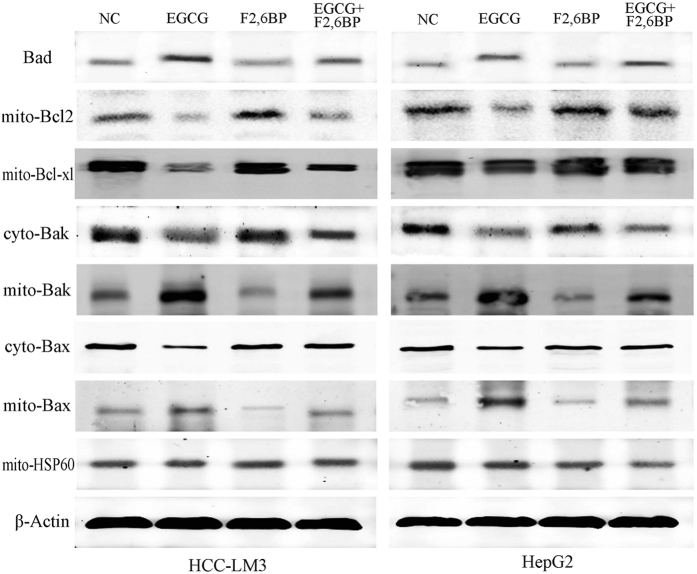
EGCG-induced effects on apoptotic regulatory proteins in HCC cells and reversal of these effects by F2,6BP. Western blot analysis of the expression of Bad, mito-Bcl2, mito-Bcl-xl, cyto-Bax, mito-Bax, cyto-Bak, and mito-Bak in HCC cells treated as indicated. Actin and hsp-60 served as loading controls.

Our data demonstrated the involvement of PFK in EGCG-induced apoptosis; however, the specific underlying mechanism remained unclear. PFK was recently identified as a novel Bad-associated pro-apoptotic protein^13^. Our results showed that EGCG treatment promoted Bad activation, as evidenced by its increased protein expression (Fig. 7). The addition of F2,6BP reversed the effect (Fig. 7), indicating that the apoptosis regulatory effect of PFK is partly mediated by binging to Bad.

Previous studies indicated that Bad exerts its pro-apoptotic effect by regulating other Bcl-2 family proteins^47^ involved in the control of apoptosis through the regulation of cytochrome c release^48^,^49^. As shown in Fig. 7, EGCG induced the translocation of Bax and Bak from the cytosol to mitochondria and downregulated Bcl-2 and Bcl-xl in mitochondria. F2,6BP effectively reversed the EGCG-induced enhancement of the pro-apoptotic effect of Bax and Bak and the decreased anti-apoptotic effect of Bcl-2 and Bcl-xl in HCC-LM3 and HepG2 cells (Fig. 7), suggesting that other Bcl-2 family proteins are involved in EGCG-induced apoptosis.

### EGCG enhanced sorafenib induced cell growth inhibition in sorafenib-resistant HCC cells

**Figure 8:**
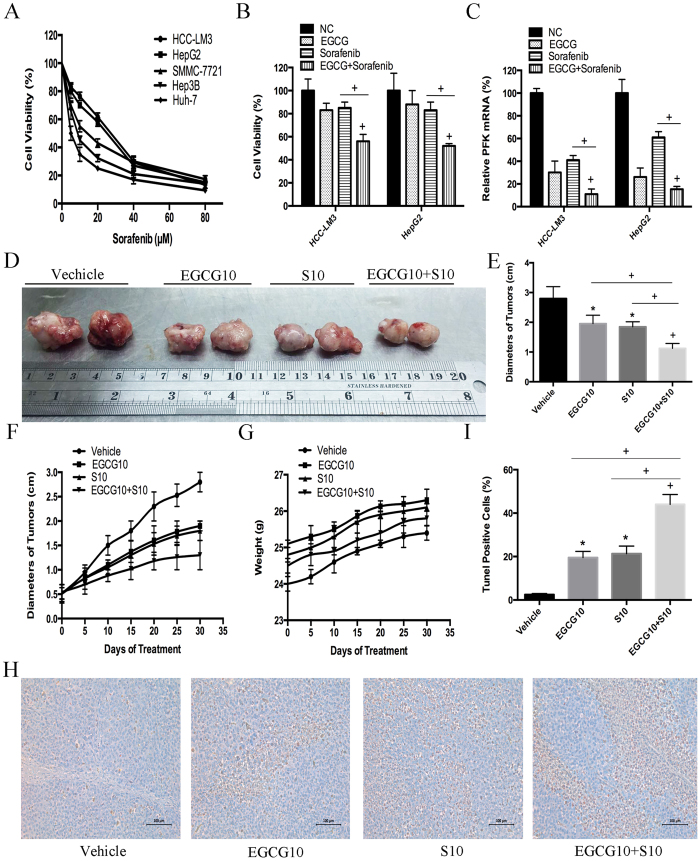
EGCG increased sorafenib induced cell growth inhibition in both sorafenib-resistant HCC cells and nude mice bearing xenograft tumors. (**A**) After sorafenib (5–80 μM) treatment for 24 h, HCC cells (5 × 10^4^) were harvested and analyzed for cell growth inhibition using the CCK-8 assay. (**B**,**C**) HCC-LM3 and HepG2 cells were exposed to EGCG (25 μM), sorafenib (5 μM), or combination treatment for 24 h, cell proliferation was analyzed using the CCK-8 assay (**B**), and PFK mRNA expression was tested using qRT-PCR (**C**). (**D**–**I**) In a xenograft mouse model, mice were treated with EGCG (10 mg/kg BW per day) alone, sorafenib (10 mg/kg BW per day) alone, or combination treatment for 30 days. At the time points indicated, the following measurements were performed: diameter of tumors (**D,E**), changes in the diameter of tumors (**F**), changes in body weight (**G**), and the percent of TUNEL-positive tumor cells (**H**,**I**). Plotted values represent the mean ± standard error of three independent experiments (n = 3) (*P < 0.05; ^+^P < 0.01).

Sorafenib is the only effective anti-HCC drug in clinical practice, although the development of resistance greatly limits its clinical efficacy. Emerging evidence suggests that a dysregulated Warburg-like glucose metabolism contributes to sorafenib-resistance in HCC cells, and the combination of chemotherapeutic drugs with tumor glycolysis inhibitors may represent a promising strategy to overcome drug resistance^50^,^51^. Therefore, we investigated the effect of combination treatment with EGCG and sorafenib on cell metabolism and cell proliferation in HCC cell lines. The five HCC cell lines tested showed different cell viability rates after exposure to sorafenib (Fig. 8A). Among them, the two high-glycolytic HCC cell lines, HCC-LM3 and HepG2, showed the highest IC_50_ for sorafenib (27.50 and 25.16 μM, respectively), which was consistent with the conclusions of the previous reports^50^,^51^. These cells were regarded as typical sorafenib-resistant HCC cells. Cell viability was significantly suppressed in response to combination treatment with EGCG (25 μM) and sorafenib (5 μM) compared with EGCG or sorafenib alone in both HCC-LM3 and HepG2 cells (P < 0.01; Fig. 8B). To investigate the possible mechanism, PFK mRNA expression was tested. Compared with EGCG or sorafenib alone, combination treatment further inhibited PFK expression in both HCC-LM3 and HepG2 cells (P < 0.01; Fig. 8C). This indicated that the combination treatment enhanced the proliferation inhibition effect potentially by suppressing PFK expression.

To explore the effects of combination treatment with sorafenib and EGCG *in vivo*, a mouse xenograft model was established using HCC-LM3 cells. Saline, sorafenib (10 mg/kg)^51^,^52^, EGCG (10 mg/kg)^53^, and sorafenib (10 mg/kg) + EGCG (10 mg/kg) were used for the *in vivo* experiments.

Mice treated with EGCG (10 mg/kg/ BW/day) or sorafenib (10 mg/kg/BW/day) alone showed a significantly smaller tumor diameter than untreated mice after 30 days of treatment (1.95 ± 0.28, 1.85 ± 0.15 *vs*. 2.81 ± 0.41, P = 0.036, P = 0.022, respectively; Fig. 8D,E). Alternatively, EGCG combined with sorafenib significantly suppressed tumor size compared with sorafenib alone (1.13 ± 0.09 *vs*. 1.85 ± 0.15, P < 0.001; Fig. 8D,E). The results of dynamic observations of the anti-tumor effects during 30 days of treatment showed the same pattern (Fig. 8F), while no significant body weight loss was observed during the 30 days of treatment (Fig. 8G). Compared to the sorafenib alone, combination treatment with EGCG and sorafenib significantly increased the rate of apoptosis (44.14 ± 5.13% *vs*. 21.33 ± 3.67%, P < 0.0001; Fig. 8H,I). Taken together, these results indicated that EGCG enhanced sorafenib induced cell growth inhibition in aerobic glycolytic HCC cell lines, and combination treatment with both reagents significantly inhibited tumor growth and induced apoptosis compared with sorafenib alone.

## Discussion

The high energy requirements of tumors, together with their low energy production, leads to an increased metabolic rate^54^. Glycolysis generates ATP more rapidly than OXPHOS, and this offers a selective advantage to rapidly growing tumor cells^3^,^4^. Furthermore, the activity of glycolysis pathway enzymes is increased in malignant cells^55^,^56^. Because of these changes, a reduced amount of pyruvate is available to mitochondria, resulting in a low oxidation rate via the tricarboxylic acid cycle^55^. These observations are consistent with our findings in human hepatoma cells, in which even under normoxic conditions, 80% of examined HCC cells displayed aerobic glycolysis, as indicated by increased lactate production and glucose uptake and decreased oxidative capacity (Fig. 1). Glycolysis has been proposed as a promising target for the development of new antitumor drugs based on the fact that anticancer agents need to be selective against tumor cells without affecting normal cells. On the basis of our results, we proposed a model for the molecular mechanism of EGCG-induced apoptosis in HCC-LM3 and HepG2 cells. Our experimental findings indicated that EGCG, a naturally occurring compound with glycolysis inhibitory activity (Fig. 2), has antitumor activity through the induction of apoptosis in hepatoma cells both in culture (Fig. 3) and in tumor-bearing mice (Fig. 8), demonstrating the correlation between glycolysis and apoptosis. However, at the lowest concentration of 25 μM, EGCG reduced glucose uptake and lactate levels, whereas it had no significant effect on suppressing proliferation or apoptosis at doses below 50 μM, suggesting that glycolysis inhibition by EGCG in high-glycolytic HCC cells occurs before the inhibition of cell growth and apoptosis induction. This is in agreement with previously reported findings^51^.

PFK is a key rate-limiting enzyme in the glycolytic pathway. In the present study, we confirmed that the increased rate of glycolysis in tumor cells is associated with increased activity of PFK (Fig. 1), which is consistent with previous findings^37^. Recent publications show that the activity of PFK can be regulated by physiological (lactate^36^, citrate, F2,6BP^57^, serotonin^58^ and insulin^59^) and non-physiological (acetylsalicylic acid (ASA), salicylic acid (SA)^37^, clotrimazole^39^, and resveratrol) ligands. This led us to hypothesize that the effects of EGCG on the induction of apoptosis through the inhibition of glycolysis could be mediated (non-exclusively, but partially) by the modulation of PFK. Our results showed that of all the glycolytic enzymes analyzed, mRNA and protein levels of PFK decreased the most in both HCC-LM3 and HepG2 cells treated with EGCG (Fig. 2C–E). EGCG had a similar effect on the activity of PFK (Fig. 2F,G), and these effects were dose-dependent. We also demonstrated that the reagent directly inhibited PFK, altering its quaternary structure (Fig. 2H,I) through a mechanism common to other modulators of the enzyme^36^,^40^. Recent studies showed the effects of four bifunctional 6-phosphofructo-2-kinase/fructose-2,6-bisphosphatases (PFKFB1-4) on PFK activity^60^,^61^; therefore, it would be of interest to determine whether EGCG functions through the regulation of PFKBs to indirectly inhibit PFK.

PFK was recently shown to be involved in the control of apoptosis^13^. In the present study, citrate, a PFK activity inhibitor, showed an enhancement effect with EGCG on glycolysis, proliferation inhibition, and apoptosis induction (Fig. 4). In addition, F2,6BP, an activator of PFK, significantly attenuated EGCG-induced apoptosis, especially mitochondrial apoptosis, in aerobic glycolytic HCC cells, as indicated by the inhibition of the loss of Δψm, the increase of activated caspases, and the release of cytochrome c (Fig. 5). Nevertheless, despite the effect of F2,6BP on reversing EGCG-induced apoptosis in HCC-LM3 and HepG2 cells, apoptosis induced by siRNA knockdown of PFK in the presence or absence of EGCG was not reversed by F2,6BP (Fig. 6), suggesting a direct involvement of PFK in EGCG-induced cytotoxicity in human hepatoma cells.

**Figure 9:**
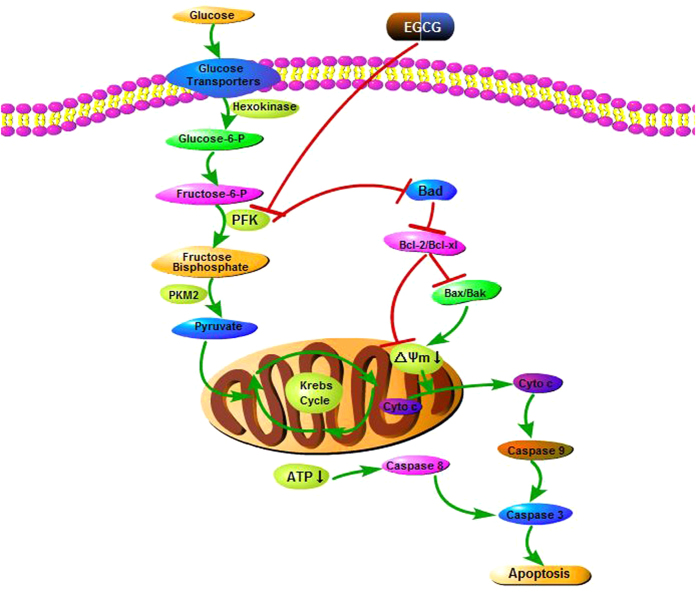
Mode of EGCG-induced apoptosis in HCC cells. Glucose uptake by GLUT2 and aerobic glycolysis by PFK are inhibited with EGCG, resulting in the suppression of ATP production. This leads to mitochondrial membrane potential breakdown and causes direct caspase-8 activation, resulting in caspase-3 activation and cell death. The interaction of EGCG and PFK frees Bad, which interacts with the anti-apoptotic Bcl-2 family proteins Bcl-2 and Bcl-xl to relieve their inhibition of pro-apoptotic proteins Bax and Bak. Oligomerization and activation of Bax and Bak leads to mitochondrial outer membrane permeabilization. Permeabilization of the mitochondrial membranes leads to the release of cytochrome c into the cytoplasm and activation of caspase-9 and caspase-3, resulting in nuclear DNA fragmentation and cell apoptosis.

This leads to question the role of PFK in apoptosis. A recent study suggested that at the onset of apoptosis, PFK binds to and suppresses the activity of the pro-apoptotic protein Bad in IL-3-dependent hematopoietic cells^13^. The results of our study in HCC-LM3 and HepG2 cells showed that EGCG unregulated Bad expression (Fig. 7). We speculated that EGCG might interact with PFK/Bad complexes by binding to PFK, resulting in free Bad. F2,6BP effectively antagonized the downstream products of Bad induced (Bax and Bak) or suppressed (Bcl-2 and Bcl-xl) by EGCG (Figs 7 and 9). Therefore, the PFK/Bad complex and factors downstream of Bad may be key targets for PFK-related apoptosis. However, the specific stereochemical structure of the PFK/Bad complex and the mechanism underlying the effect of PFK on apoptosis in HCC cells in the presence or absence of EGCG remain to be elucidated.

HCC is an incurable disease^62^,^63^,^64^,^65^,^66^,^67^, the incidence and mortality of which are increasing yearly^68^,^69^,^70^,^71^,^72^,^73^,^74^. Sorafenib is the only drug approved for the treatment of HCC^50^,^51^,^52^. However, the low rate of tolerance to sorafenib among HCC patients limits its benefits^75^. Therefore, new chemotherapeutic options are urgently needed. The bioenergetic properties of cancer cells have been correlated to anticancer drug resistance^76^,^77^. Glycolysis, which is a potential predictive biomarker of sorafenib resistance^50^, is used to overcome sorafenib resistance. Resveratrol attenuates sorafenib resistance via glycolysis^51^. Since EGCG is able to inhibit glycolysis in aerobic glycolytic HCC cells (Fig. 2), we hypothesized that EGCG might enhance the effect of sorafenib. Two high-glycolytic HCC cells, HCC-LM3 and HepG2, which also showed the highest IC_50_ for sorafenib among all the HCC cells examined (Fig. 8A), were selected for further research. The results of our study showed that EGCG enhanced sorafenib-induced cell growth inhibition in aerobic glycolytic HCC cells (Fig. 8B). EGCG, when used in combination with sorafenib, enhanced the effects of sorafenib on reducing tumor size and increasing apoptosis in HCC-transplanted nude mice (Fig. 8A–H), which was consistent with the conclusions of previous reports^50^,^51^. Our results provide preclinical evidence of the potential value of EGCG for the treatment of HCC. Although the *in vivo* doses of the reagent used in our study were high, EGCG is not toxic at higher doses in mice or humans^23^,^78^. EGCG, as a chemosensitizer, ameliorates the deleterious side effects of chemo- and radiotherapy^79^. Our findings provide a basis for the development of novel strategies for the treatment of sorafenib-resistant HCC patients.

To the best of our knowledge, the present study is the first to show the antitumor effects of EGCG mediated by the inhibition of glycolysis in aerobic glycolytic HCC cells. EGCG acts by directly suppressing PFK activity as demonstrated by two findings: first, EGCG transformed the oligomeric structure of PFK into its inactive form; and second, apoptosis induced by PFK siRNA knockdown was not further enhanced by EGCG. Although the PFK allosteric activator reversed EGCG-induced apoptosis, it had no effect on PFK siRNA knockdown HCC cells. In addition, we showed for the first time that EGCG enhanced the anti-tumor effect of sorafenib, which indicated that the prognosis of sorafenib-resistant HCC patients could be improved through the combination treatment proposed in the present study.

## Materials and Methods

### Reagents and cell culture

EGCG, trisodium citrate dihydrate, and F2,6BP were purchased from Sigma-Aldrich (St. Louis, MO, USA). For cell treatment, the compounds were dissolved in phosphate buffer saline (PBS) and then diluted in media with 10% fetal bovine serum (FBS; Hyclone, Logan, UT, USA) before use. Sorafenib tosylate was purchased from Selleck (Selleck Chemicals, Shanghai, China), and was dissolved in dimethyl sulfoxide (Sigma-Aldrich, St. Louis, MO, USA).

All cultured cells were purchased from the Chinese Academy of Sciences Committee Type Culture Collection cell bank. HCC-LM3, Huh-7, HepG2, Hep3B, SMMC-7721, and LO2 cells were maintained in Dulbecco’s modified Eagle’s Medium (DMEM) with high glucose (Hyclone) supplemented with 10% FBS. QSG-7701 was cultured in RPMI-1640 with 10% FBS at 37 °C in a humidified atmosphere of 5% CO_2_.

[^32^P]H_2_PO_4_ was purchased from Instituto de Pesquisa em Energia Nuclear (IPEN, Brazil) and used to prepare [γ-^32^P]ATP as previously described^37^. PFK was purified from rabbit skeletal muscle^80^, with the modification introduced by Kuo *et al*.^81^. Muscle homogenates^58^ and erythrocytes membrane^82^ were prepared as described previously.

### Cell proliferation analysis

HCC cells were cultured with the indicated concentrations of EGCG before the addition of 10 μl CCK-8 solution (Peptide Institute Inc., Osaka, Japan) to each well. The plate was maintained in the incubator for 2 h. The absorbance was measured at 450 nm using a microplate reader. The half maximal inhibitory concentration (IC_50_) values were calculated using CompoSyn software using the absorbance data obtained with a microplate reader Model 680 (BIO- RAD, Hercules, CA, USA).

### Cell cycle analysis

Cells were treated with EGCG and collected. Then, cells were fixed with cold 70% ethanol overnight. After washing once with PBS, cells were stained with propidium iodide (PI; BD BioSciences, San Jose, CA, USA) and analyzed on a BD FASCCanto II flow cytometer (Cytomics FC500; Beckman Coulter, Fullerton, CA, USA).

### Apoptosis analysis

HCC cells were cultured in six-well plates and treated as indicated. After 24 h, the cells were centrifuged, washed twice with PBS, and mixed in 100 μl of 1× binding buffer (10 mM HEPES/NaOH, pH 7.4, 140 mM NaCl, 2.5 mM CaCl_2_). After culturing for 15 min at room temperature in Annexin-V/PI (BD Biosciences) double staining liquid, the cells were examined in a flow cytometer (Cytomics FC500). The percentage of apoptotic cells was calculated using ModFitLT software.

### JC-1 staining to measure mitochondrial membrane potential

Cells were plated on coverslips with different treatments overnight. After staining with 10 μg/ml JC-1 (JC-1 Mitochondrial Membrane Potential Detection Kit; Invitrogen) for 15 min in the incubator (37 °C, 5% CO_2_), cells were rinsed with PBS twice to remove the non-specific background staining. Cells were analyzed using a flow cytometer (Beckman Coulter).

### 2-DG uptake, lactate production, and O_2_ consumption

Cells were washed twice with the uptake buffer (140 mM NaCl, 2 mM KCl, 1 mM KH_2_PO_4_, 10 mM MgCl_2_, 1 mM CaCl_2_, 5 mM glucose, 5 mM L-alanine, 5 mM indomethacin, and 10 mM HEPES/Tris, pH 7.4) and cultured in uptake buffer containing 1 μCi/ml [^3^H]-2-DG at 37 °C for 30 min. Then, the cells were solubilized with 0.1% sodium dodecyl sulfate and the radioactivity was calculated using a liquid scintillation counter. The radioactivity counts for each sample were normalized to the protein content and corrected for the zero-time uptake per mg protein. Lactate levels were measured by a fluorometric assay (BioVision, Milpitas, CA, USA) according to the manufacturer’s protocol. O_2_ consumption was tested using the 110 Fiber optic oxygen monitor (Instech, Plymouth Meeting, PA, USA). Approximately 5 million cells were collected in 500 μl of media and cultured at 37 °C. Results were obtained from three independent experiments and expressed as nmol O_2_/million cells/min.

### Reverse transcription (RT)-PCR and quantitative real time (qRT)-PCR

The TRIzol reagent was used to extract total RNA according to the manufacturer’s protocol. SYBR Green quantitative RT-PCR was performed to determine gene expression level using a 7900HT fast real-time PCR system (Applied Biosystems, CA, USA), according to the protocols provided with the SYBR Premix EX Taq (TaKaRa Biotechnology, China). The levels of the target gene were normalized those of β-actin.

### Protein extraction and western blotting

The cytosolic and mitochondrial fractions were separated and purified from HCC cells using a Mitochondrial Isolation Kit (Pierce, Rockford, IL, USA) according to the manufacturer’s protocol^68^. Total cellular proteins were extracted using radio-immunoprecipitation assay (RIPA) buffer (Sigma-Aldrich) containing protease inhibitors.

The samples were then resolved by sodium dodecyl sulfate-polyacrylamide gel electrophoresis and transferred to polyvinyl difluoride membranes. The membranes were sequentially blocked in PBS containing 0.1% Tween 20 (PBST) with 5% non-fat milk and probed with primary antibodies (Cell Signaling Technology, Danvers, MA, USA). Membranes were then washed with PBST three times and incubated with the secondary antibody horseradish peroxidase-conjugated anti-rabbit or anti-mouse IgG (1:2000) for 1 h at room temperature. Finally, the membranes were washed three times and scanned using the Odyssey two-color infrared laser imaging system (fluorescence detection). Molecular sizes were determined by comparison with the prestained molecular weight markers.

### Plasmids and transfection assays

PFK expression in liver cancer cells was ablated with small interfering RNAs (siRNAs). The target sequences of PFK siRNA (5′-GGTGCCCGTGTCTTCTTTGT-3′, 5′-AGCGTTTCGATGATGCTTCAG-3′, 5′-AGCTGCCTACAACCTGGTGA-3′) were selected to suppress PFK gene expression, and PFK scramble siRNAs (scRNAs) were used as controls. All plasmid sequences were confirmed by DNA sequencing. The siRNAs were transfected into cells using Lipofectamine™ 2000 following the manufacturer’s instructions. The transduction efficiency was measured by western blotting. The sorted cells were then characterized and used in further assays.

### Spectrophotometric assay for PFK activity

PFK activity was assayed in a medium containing 50 mM Tris–HCl (pH 7.4), 5 mM MgCl_2_, 5 mM (NH_4_)_2_SO_4_, 1 mM F6P, 1 mM ATP, 0.5 mM NADH, 2 mU/ml aldolase, 2 mU/ml triosephosphate isomerase, 2 mU/ml α-glycerophosphate dehydrogenase, and 50 μg/ml of protein in a final volume of 200 μl as previously described^37^. Other reagents used are indicated for each experiment. The reaction was started by the addition of protein, and NADH oxidation was followed by measuring the decrease in absorbance at 340 nm in a microplate reader.

### Radioassay for PFK activity

PFK activity was measured using a previously described method^83^ with modifications^84^ in a medium containing 50 mM Tris-HCl (pH 7.4), 5 mM MgCl_2_, 5 mM (NH_4_)_2_SO_4_, 1 mM F6P, 1 mM [γ-^32^P]ATP, and 1 mg/ml purified PFK. The reaction was initiated by the addition of the enzyme and aliquots were withdrawn at increasing reaction times and transferred to tubes containing activated charcoal in 0.1 M HCl to interrupt the reaction and adsorb the unreacted [γ-^32^P] ATP. After centrifugation, [γ-^32^P] ATP-adsorbed activated charcoal was pelleted, and aliquots of the supernatant containing [γ-^32^P] F1,6BP were withdrawn and the radioactivity evaluated in a liquid scintillation counter. Blanks without F6P were run in parallel and the radioactivity produced was subtracted from the data to correct for ATP hydrolysis. The amount of F1,6BP (nmol) measured in each condition was plotted against reaction time, and the linear coefficient of the curve was used to express PFK activity (mU).

### Intrinsic fluorescence measurements

PFK intrinsic fluorescence measurements were performed as described previously^85^ using the same conditions described for the radioassay. Excitation wavelength was fixed at 280 nm and fluorescence emission was scanned from 300 to 400 nm. The center of mass of the intrinsic fluorescence spectra (cm) was calculated using the following formula:


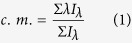


where λ is the wavelength and I_λ_ is the fluorescence intensity at a given λ. The center of mass is used to evaluate the oligomeric state of PFK because in the dissociated enzyme, the tryptophan residues are exposed to the aqueous milieu, and the fluorescence emitted by these tryptophans is of lower energy. Consequently, the center of mass of a population of tetramers is smaller than the parameter measured for a population of dimers, as demonstrated in many recent publications^39^,^40^.

### Animal experiments

Four-week-old male athymic BALB/C nu/nu mice with free access to water and food were housed in a standard animal laboratory with a 12-h light-dark cycle and constant environmental conditions. All experiments were performed in accordance with ethical standards and in compliance with the Declaration of Helsinki, and according to national and international guidelines. The study was approved by the Animal Care and Use Committee of Shanghai Tongji University. Serum-free culture medium (300 μl) containing HCC-LM3 cells (5 × 10^6^) was subcutaneously injected into the upper flank region of 20 mice. When the tumor volume was approximately 100 mm^3^, the animals were randomly divided into four groups (saline, EGCG, sorafenib, and EGCG + sorafenib). Sorafenib was dissolved in an oral vehicle containing Cremophor EL (Sigma-Aldrich), 95% ethanol, and water in a ratio of 1:1:6 as described previously^52^, and orally administrated at a dose of 10 mg/kg by gavage daily for 30 days. EGCG was dissolved in saline and administrated by intraperitoneal injection once daily for 30 days^53^. Mice in the vehicle group received oral and intraperitoneal vehicles; mice in the EGCG group received oral vehicle and intraperitoneal EGCG; mice in the sorafenib group received intraperitoneal vehicle and oral sorafenib; and mice in the EGCG + sorafenib group received intraperitoneal EGCG and oral sorafenib. During the procedure, tumor size and body weight of mice were measured every 5 days. At the end of the experiment, mice were killed and tumors were resected and imaged using a high-definition digital camera.

### Terminal deoxynucleotidyl transferase (TdT) dUTP nick-end labeling (TUNEL) assay

Apoptosis of tumor tissues was assessed using the TUNEL assay. Paraffin-embedded sections (5 μm) were cut and mounted on glass slides. The sections were deparaffinized and then digested with 20 μg/ml proteinase K (Sigma-Aldrich) for 15 min at room temperature. The slides were washed four times in distilled water for 2 min, incubated with 2% hydrogen peroxide in PBS for 5 min at room temperature, washed with PBS twice, and immersed in TdT-containing buffer for 15 min to prepare digoxigenin-binding sites. An anti-digoxigenin antibody fragment carried a conjugated reporter enzyme (peroxidase) to the reaction sites, and then localized peroxidase generated an intense signal from the chromogenic substrate diaminobenzidine. The counterstain was methyl green.

### Statistical analysis

Statistical analysis was performed using a two-tailed unpaired Student’s t-test and SPSS 17.0 software. Quantitative data are representative of at least three independent experiments. Values of *P < 0.05 and ^+^P < 0.01 were considered statistically significant.

## Additional Information

**How to cite this article**: Li, S. *et al*. *In vitro* and *in vivo* study of epigallocatechin-3-gallate-induced apoptosis in aerobic glycolytic hepatocellular carcinoma cells involving inhibition of phosphofructokinase activity. *Sci. Rep*. **6**, 28479; doi: 10.1038/srep28479 (2016).

## Supplementary Material

Supplementary Information
