# Supplementary material for: In vitro and in vivo study of epigallocatechin-3-gallate-induced apoptosis in aerobic glycolytic hepatocellular carcinoma cells involving inhibition of phosphofructokinase activity
Source: Sci Rep. 2016 Jun 28;6:28479. doi: 10.1038/srep28479 (PMC4923908; doi:10.1038/srep28479)
Supplement: Supplementary Information [file srep28479-s1.pdf]

Supplementary information

# *In vitro* and *in vivo* study of epigallocatechin-3-gallate-induced apoptosis in aerobic glycolytic hepatocellular carcinoma cells involving inhibition of phosphofructokinase activity

Sainan Li[1], Liwei Wu[1], Jiao Feng[1], Jingjing Li[1], Tong Liu[1], Rong Zhang[1,2], Shizan Xu[1,2], Keran Cheng[1,3], Yuqing Zhou[1,3], Shunfeng Zhou[1,3], Rui Kong[1,3], Kan Chen[1], Fan Wang[1], Yujing Xia[1], Jie Lu[1], Yingqun Zhou[1], Weiqi Dai[1], Chuanyong Guo[1]

[1]Department of Gastroenterology, Shanghai Tenth People's Hospital, Tongji University School of Medicine, Shanghai 200072, China. [2]The Shanghai Tenth Hospital School of Clinical Medicine of Nanjing Medical University，Shanghai 200072, China. [3]The School of Medicine of Soochow University，Suzhou 215006, China.

Corresponding authors: Weiqi Dai and Chuanyong Guo. Tel: +86-21-66302535. Fax: +86-21-66303983

Email: dai_yue@163.com; guochuanyong@hotmail.com

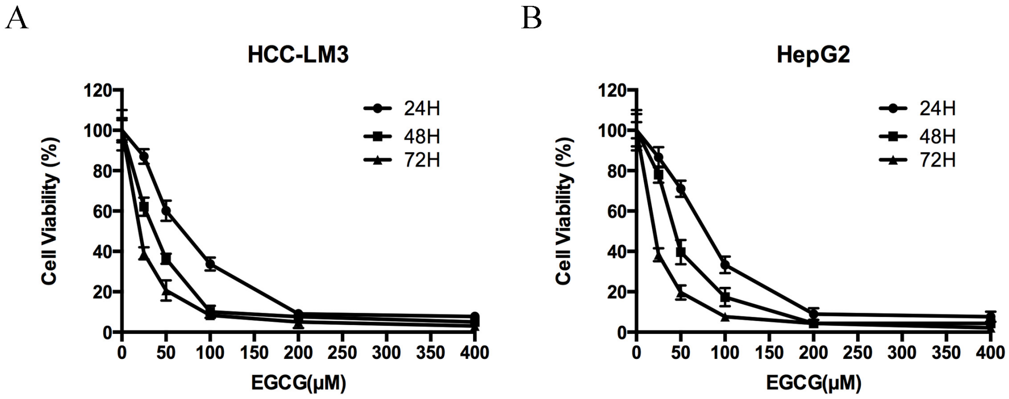


**Supplementary Figure S1: Effects of EGCG on HCC cell proliferation inhibition.** (**A** and **B**) HCC cells were cultured with or without EGCG (25, 50, 100, 200 and 400 μM) for 24, 48 and 72 h, and cell viability was examined using the CCK-8 method. Plotted values represent the mean ± standard error of three independent experiments (n = 3).
